# Ameloblastin Amphipathic Helix Motif mediates Ameloblast Polarization and Prismatic Enamel Formation via a RhoA Signaling Pathway

**DOI:** 10.21203/rs.3.rs-8492927/v1

**Published:** 2026-01-28

**Authors:** Gayathri Visakan, Rucha Arun Bapat, Jing Cai, Ethan Trevor Suwandi, Derk Joester, Natalie C Kegulian, Edwin Sarkisians, Marziyeh Aghazadeh, Simon Webster, Janet Moradian-Oldak

**Affiliations:** 1. Center for Craniofacial Molecular Biology, Herman Ostrow School of Dentistry of USC, University of Southern California, Los Angeles, CA, USA.; 2. Department of Materials Science and Engineering, Northwestern University, Evanston, IL, USA.

## Abstract

Ameloblastin (Ambn) is a tooth-specific multifunctional protein crucial for enamel biomineralization and its prismatic structure. To examine the function of the evolutionarily conserved cell-binding Ambn amphipathic helix (AH) motif, we deleted the hydrophobic residues within Ambn AH motif in genetically engineered mice. Enamel in the homozygous (*Ambn*^ΔL76-P86^) mutants had normal thickness but was hypo-mineralized and lacked prismatic structure. Micro-CT analysis using conventional neural network revealed loss in mineral density and a delay in the initiation of secretory stage of amelogenesis. Ameloblasts in the mutants were stunted and exhibited loss of cell polarity, as demonstrated by the mis-localization of Pard3, Claudin-1 and GM130 immunosignals. In the *Ambn*^ΔL76-P86^ mutants, a loss of Ambn-ameloblast distal membrane interaction was observed, with nuclear localization of β-catenin and p-Smad2/3, and a decrease in RhoA immunolabeling intensity. Our study demonstrates a novel signaling role of Ambn AH motif in mediating cell polarization and forming enamel prismatic structure.

## Introduction

Dental enamel is a highly mineralized tissue that is entirely epithelial in origin^1^. The molecular interactions that govern the establishment of terminally differentiated ameloblasts are tightly controlled, with reciprocal interactions between the epithelium and the underlying mesenchyme.^2^ Evolutionarily, prismatic enamel emerged about 200 million years ago in the late Triassic period.^3,4^ With the evolution of mammals, enamel structural complexity and hierarchy increased, with a resultant arrangement of calcium hydroxyapatite mineral bundles into a highly ordered hierarchical structure comprising prisms (rods) and inter-prisms (inter-rods).^5-7^

Ameloblasts are a uniquely polarizing cell type with their Golgi positioning towards the basement membrane (away from the anatomical apical membrane) during the presecretory stage of amelogenesis. This peculiar phenomenon has been described as a reversal of polarity.^8-10^ In mouse, the basement membrane is degraded just prior to the onset of secretory stage, and the anatomical base serves as the functional apex through which secretion of proteins commences.^11^ While the structural complexity of mammalian prismatic enamel has often been correlated with ameloblast morphological features,^12^ including differential secretion along the surfaces of the Tomes’ Process (TPs) patterning enamel prisms and inter-prisms^13-15^ molecular mechanisms that govern such correlation has not been well studied.

Ameloblastin is the second most predominant EMP after amelogenin, and in recent years has emerged as a multifunctional protein involved in extracellular matrix mineralization, cell adhesion and differentiation.^16^ The exon 5-encoded region of ameloblastin is part of a recently identified multitargeting domain in the N-terminal region. We have demonstrated that this domain encompasses the evolutionarily conserved cell binding amphipathic helix forming (AH) motif^17,18^ in addition to the Ambn self- assembly^19^ and Ambn-Amel co-assembly motif.^20^ Genetically engineered animal models of *Ambn* truncation and deletion that result in a fundamental lack of true enamel^21-23^ and *Amelogenesis Imperfecta* involving *AMBN* ^23,24^ further highlight the indispensability of this protein during amelogenesis.

Among the species with functional prismatic enamel, there is a high degree of conservation of residues that primarily reside in the N-terminal region of ameloblastin.^25^ Within the N-terminal region of Ambn exon 5 is the AH motif, spanning from residues 69 - 86 of mouse Ambn.^17^ Amphipathic helices were first discovered in the context of apolipoproteins and antimicrobial peptides and are classical cell binding motifs.^26,27^ We have discovered that Ambn AH motif mediates direct cell membrane interaction with ameloblasts and is the bioactive region that can promote the elongation and polarization of ameloblast cell lines in 3D cell culture.^28-30^ We reported that within the EMPs, the AH motif is unique to ameloblastin,^28^ and within vertebrates, only ameloblastin from mammals contains the AH motif.^31^ Subsequently, by evolutionary analysis of enamel structure and ameloblastin sequence from 53 vertebrates we determined that the AH motif and prismatic enamel co-evolved in mammals.^31^

Here, using a targeted deletion of hydrophobic residues within the AH motif, we present a genetically engineered animal model for supporting the hypothesis that a functional AH motif is necessary for the establishment of a prismatic enamel structure. It has generally been accepted that ameloblast cell shape is what patterns the matrix structure and in turn the mineral organization.^5,32,33^ We demonstrate here that the highly elongated and polarized ameloblast morphology, a pre-requisite for matrix secretion and hierarchical patterning, arises consequent to interactions of the Ambn AH motif with the distal ameloblast cell membrane. In our newly designed mutants, we observe a peculiar phenotype where full-thickness enamel is formed, albeit with a loss of mineral density and a lack of prismatic organization. Unlike previous models, one copy of the deletion was sufficient to generate a phenotype. Importantly, these observations mirror findings from a case of *Amelogenesis Imperfecta* resulting from a truncation of *AMBN* within the AH motif region, further adding to the clinical relevance of this model.^24^ Finally, using this model we reinforce the concept of multifunctionality of Ambn, with a selective disruption of ameloblastin-ameloblast interactions.

## Results

### Design and validation, and characterization of genetically engineered *Ambn*^ΔL76-P86^ mutant mice

**Figure 1.**
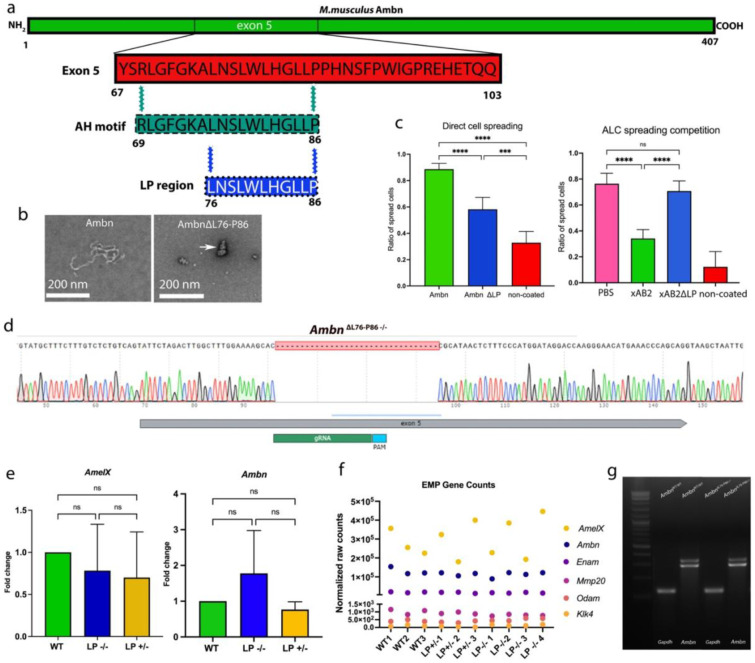
Ameloblastin LP region mutant, *Ambn*^ΔL76-P86^, animal design and validation. (**a**) Schematic representation of exon 5 and the LP region, spanning from Lys76 - Pro86 within mouse ameloblastin AH motif. (**b**) Transmission electron microscopy analysis of recombinant mouse Ambn, and recombinant mouse AmbnΔL76-P86 mutants. (**c**) ALC direct spreading assay on plates coated with recombinant mouse Ambn and AmbnΔL76-P86 mutant (left). ALC spreading competition assay with full-length (xAB2) and mutant peptide (xAB2ΔL76-P86) corresponding to the ameloblastin multitargeting region ^34^ (right). (**d**) Sanger sequencing analysis of genetically engineered *Ambn*^ΔL76-P86−/−^ animal showing the successful in-frame thirty-three base-pair deletion corresponding to Ambn Lys76 - Pro86. (**e**) qPCR analysis of *AmelX* (left) *Ambn* (right) and fold change comparison from P5 mandibular molar enamel organs of *Ambn*^WT/WT^, *Ambn*^ΔL76-P86 +/−^ and *Ambn*^ΔL76-P86 −/−^. (**f**) RNA sequencing fold change analysis of major enamel matrix protein genes. (**g**) Genotyping results of *Ambn* transcript (isoform) analysis. * *p* < 0.05; **p < 0.01, *** *p* < 0.001, n/s- not significant.

To specifically interrupt the cell-binding domain of Ambn, we selected a targeted eleven-amino-acid residue deletion, spanning from Lys76 to Pro86 of mouse Ambn. This region is thereafter referred to as the LP region (blue in Figure 1a). A Color Conservation Analysis on aligned Ambn sequences corresponding to the LP region from mouse, pig and human ameloblastin showed that within the eleven amino acid residues of the LP region, nine out of eleven residues share a 100% sequence identity across the three species examined (Supplementary Figure 1a).

To support the design of the novel mutant animals; *Ambn*^ΔL76-P86^, we designed a recombinant mouse Ambn mutant protein corresponding to the deletion of LP region (Supplementary Figure 1b) and evaluated Ambn interactions. Transmission electron microscopy (TEM) and Dynamic light scattering (DLS) were used to examine the self-assembly properties of the recombinant mouse AmbnΔL76-P86 compared to wild type (WT) recombinant mouse Ambn (positive control) and recombinant Ambn Δ5 (negative control). Ribbon-like structures were observed in the recombinant Ambn samples ranging from tens to hundreds of nanometers in length (Figure 1b, left). Recombinant AmbnΔL76-P86, while heterogenous, exhibited a tendency to self-assemble and formed small fiber-like structures in the range of tens of nanometers (Figure 1b, right, white arrow). Recombinant AmbnΔ5 did not form ribbon-like structures (Supplementary Figure 1c). DLS analysis of AmbnΔL76-P86 protein solution showed a heterogeneous particle size distribution between 90-200 nm which was comparable to the WT recombinant mouse Ambn (Supplementary Figure 1d).

The effect of AH motif truncation on Ambn-ameloblast cell membrane interaction was examined *in vitro* using Ameloblast lineage cells (ALC), full-length and mutant recombinant Ambn proteins, and corresponding peptides (called xAB2)^34^ in direct cell spreading and cell spreading competition assays respectively. On average, 88.6% of ALCs cultured on recombinant WT Ambn coated plates adopted a spread morphology suggesting the presence of intact membrane interaction domain(s). In the case of recombinant AmbnΔL76-P86 mutant, disruption of the helix resulted in only 51.3-58.2% of ALCs adopting a spread morphology (*p* < 0.001) (Figure 1c, left). The loss of membrane interaction and the specificity of the LP region in mediating these cell interactions were confirmed by cell spreading competition assay using synthetic peptides (Figure 1c, right). Only 34% of cells treated with full-length peptide adopted a spread morphology on recombinant WT Ambn coated plates, suggesting that the successful xAB2 peptide-ALC membrane interaction (*p* < 0.001) was sufficient to inhibit ALC cell spreading. With mutant xAB2ΔL76-P86 as the competing peptide, 70% of ALC adopt a spread morphology on recombinant WT Ambn-coated plates (*p* < 0.001). This loss of inhibition of spreading suggests the inability of the mutant xAB2ΔL76-P86 peptide to interact with ALC membrane.

Sanger sequencing confirmed the successful generation of an in-frame deletion of thirty-three base pairs, that correspond to the *Ambn*^ΔL76-P86^ deletion, without any other off-target effects (Figure 1d).Importantly, quantitative PCR analysis revealed no significant differences between the *Ambn*^ΔL76-P86^ mutants and *Ambn*^WT/WT^ in the expression levels of *AmelX* and *Ambn* in the *Ambn*^ΔL76-P86^ mutants compared to *Ambn*^WT/WT^ (Figure 1e, left and right) (*p* > 0.05). Additionally, RNA sequencing analysis revealed no change in the expression levels of the major extracellular matrix (ECM) genes involved in amelogenesis (Figure 1f). Both *Ambn* isoforms were expressed at comparable levels in in the *Ambn*^ΔL76-P86^ mutants compared with *Ambn*^WT/WT^ (Figure 1g). These data demonstrated that interruption of the AH domain did not affect gene expression of major enamel extracellular matrix and that this is an ideal model to study the direct effect of AH motif on enamel formation.

### *Ambn*^ΔL76-P86 −/−^ mutants exhibit loss of surface texture and enamel integrity

**Figure 2.**
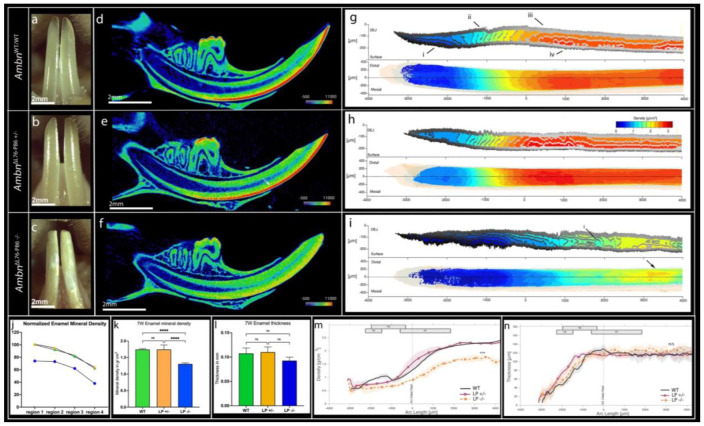
Gross enamel appearance and micro-CT analysis of 7-week-old *Ambn*^WT/WT^ and *Ambn*^ΔL76-P86^ mutant samples. (**a - c**) Optical microscopy of mouse incisors on of *Ambn*^WT/WT^ (a), *Ambn*^ΔL76-P86+/−^ (b) and *Ambn*^ΔL76-P86−/−^ (c). (**d - f**) Heatmap rendering of sagittal reconstructions of incisors of a representative *Ambn*^WT/WT^ (d), *Ambn*^ΔL76-P86+/−^ (e) and *Ambn*^ΔL76-P86−/−^ (f). (**g - i**) Rendering of iso-density surfaces of *Ambn*^WT/WT^ (g), *Ambn*^ΔL76-P86+/−^ (h) and *Ambn*^ΔL76-P86−/−^ (i). Longitudinal cross-sections (top) and facial view (bottom) of the incisor enamel overlayed with internal density iso-density surfaces. The dentin and enamel are faintly outlined in yellow and blue to show their extent. Incisors are resampled in the cylindrical coordinate system. The dotted line denotes the location of the longitudinal cross-section and the fitted circle. (**j**) Enamel mineral density plot of *Ambn*^WT/WT^ and *Ambn*^ΔL76-P86^ mutants, normalized to erupted enamel density of *Ambn*^WT/WT^. Region 4 represents earliest formed enamel and region 1 represents erupted enamel. Green hexagons: *Ambn*^WT/WT^, yellow triangles: *Ambn*^ΔL76-P86 +/−^, and blue circles: *Ambn*^ΔL76-P86 −/−^. (**k**) Absolute enamel mineral density comparison of erupted enamel from *Ambn*^WT/WT^ and *Ambn*^ΔL76-P86^ mutants (**l**) Enamel thickness comparison between *Ambn*^WT/WT^ and *Ambn*^ΔL76-P86^ mutants. (**m, n**) Comparison of incisor enamel development as a function of arc length. Incisor enamel maximum thickness (m) and mean density (n) for *Ambn*^WT/WT^ and *Ambn*^ΔL76-P86^ samples. Shaded areas in like colors indicate the 95% confidence interval. Bars represent the spatial extents of the M1, M2, and M3 molars with extremes of the bars indicating the mean and standard deviation of all samples. * *p* < 0.05; **p < 0.01, *** *p* < 0.001, n/s- not significant.

As observed by optical microscopy the enamel in *Ambn*^WT/WT^ mice appeared smooth and translucent with sharp incisal edges (Figure 2a). Incisors of *Ambn*^ΔL76-P86 +/−^ mice did not display any obvious phenotype on the labial surface except for a mild blunting of incisal edges (Figure 2b). Mandibular incisors from *Ambn*^ΔL76-P86 −/−^ mice appeared chalky white on the labial surface of the incisors (Figure 2c). The overall morphology of the incisors was altered in the *Ambn*^Δ L76-P86 −/−^ mice, with the incisors appearing shorter in comparison to *Ambn*^WT/WT^ (Figure 2 a, c).

### *Ambn*^ΔL76-P86 −/−^ mouse enamel has normal thickness but a decreased mineral density

We used both conventional micro-CT, and a newly developed conventional neural network (CNN) on high resolution micro-CT scans to examine defects in enamel mineral density and thickness (Figure 2). Qualitative analysis of incisor enamel mineral density was carried out in heatmap renderings of sagittal reconstructions (Figure 2 d - f). A clearly distinguishable enamel layer appearing red to orange on heatmap rendering (indicative of high mineral density) could be observed uniformly along the outer surface of the incisors in *Ambn*^WT/WT^ (Figure 2d). In *Ambn*^ΔL76-P86 +/−^ incisor enamel rendered yellow in patches (Figure 2e, white arrow). In *Ambn*^ΔL76-P86 −/−^ the entire incisor outer enamel layer being indistinguishable from the underlying dentin because of a decrease in mineral density when compared to *Ambn*^WT/WT^ controls (Figure 2f).

To visualize the progress of enamel densification in time and space, we inspected renderings of iso-density surfaces (Figure 2 g - i) on high resolution scans analyzed using the newly developed CNN. In *Ambn*^WT/WT^ samples, increase in mineral density starts from the dentino-enamel junction (DEJ) towards the enamel surface during secretory stage (Figure 2g, i). Approximately 500 μm after full thickness of enamel is achieved, the trend reverses with density increasing from the surface towards the DEJ. At a density of approximately 1.25 g/cm^3^, iso-density surfaces become regularly spaced, forming even columns in the DEJ-to-surface direction (Figure 2g, ii). At approximately 2.0 g/cm^3^ densification at the DEJ appears to slow down relative to the surface (Figure 2g, iii), with the densest part of the enamel forming between the surface and 50 μm (Figure 2g, iv). Heterozygous *Ambn*^ΔL76-P86+/−^ mutants appeared to follow the same trends observed in *Ambn*^WT/WT^ (Figure 2h). Homozygous *Ambn*^ΔL76-P86−/−^ mutants form demineralized regions within the body of the enamel, creating a turbulent and heterogenous density pattern (Figure 2i, i). The secretory stage density iso-surfaces appear similar to *Ambn*^WT/WT^ and *Ambn*^ΔL76-P86 +/−^. However, the transition at 1.25 g/cm^3^ is located further in the incisal direction (1000 μm) compared *Ambn*^WT/WT^ and *Ambn*^ΔL76-P86 +/−^ (−1500 to 0 μm).

In *Ambn*^WT/WT^, the incisor mineral density gradually increases moving from the earliest formed enamel at region 4 to the erupted enamel at region 1 (Figure 2j) (Supplementary Figure 2). In the *Ambn*^ΔL76-P86 −/−^ mutant, this trend between regions 4-1 was not present. The normalized mineral density on average at region 1 of the *Ambn*^ΔL76-P86 −/−^ mutant was only 74% that of *Ambn*^WT/WT^ (Figure 2j). The absolute mineral density at region 1 of *Ambn*^ΔL76-P86 −/−^ was statistically significantly lower compared to *Ambn*^WT/WT^ (n=5; *p* < 0.001) (Figure 2k). No significant change in the mineral density at region 1 was observed in *Ambn*^ΔL76-P86 +/−^ compared to *Ambn*^WT/WT^ (n=5; *p* > 0.05). Notably, measurements of incisor enamel thickness revealed that there was no significant difference between *Ambn*^WT/WT^ and both *Ambn*^ΔL76-P86^ mutants (n=5; *p* > 0.05) (Figure 2l).

Incisor enamel maximum thickness (Figure 2m) and mean density (Figure 2n) were used as indications to characterize the secretory and maturation stages on high resolution scans. The secretory stage in the both *Ambn*^ΔL76-P86^ mutants was delayed approximately 800 μm incisal of *Ambn*^WT/WT^ (Figure 2m). All genotypes displayed a sigmoidal mean density curve along the arc of the incisor (Figure 2n). In the homozygous *Ambn*^ΔL76-P86 −/−^ samples maturation stage was delayed and elongated between −500 to 2500 μm, reaching a hypo-mineralized final density of approximately 1.73 g/cm^3^.

### Prismatic enamel microstructure is lost in *Ambn*^ΔL76-P86^ mutants

**Figure 3.**
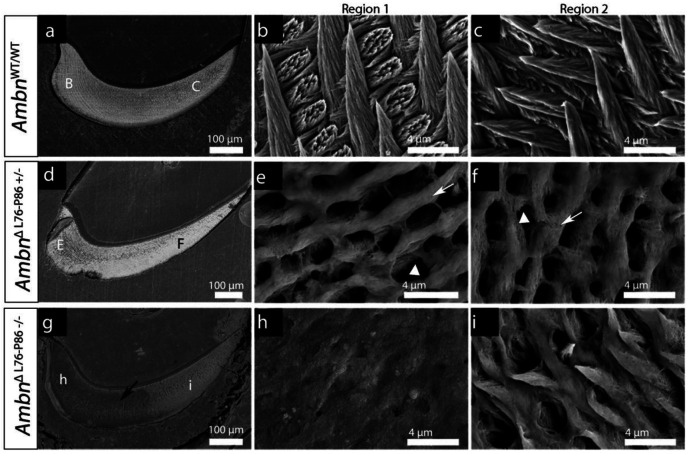
Backscattered scanning electron microscopy of enamel ultrastructure in seven-week-old *Ambn*^WT/WT^ (top panel) and *Ambn*^ΔL76-P86^ mutants (middle and bottom panels). (**a, d, g**) Low resolution images from incisor cross sections corresponding to erupted enamel. (**b, e, h**) High resolution images taken from region 1 of the incisor cross section in *Ambn*^WT/WT^ (b), *Ambn*^ΔL76-P86 +/−^ (e) and *Ambn*^ΔL76-P86 −/−^ (h). (**c, f, i**) High resolution images taken from region 2 of the incisor cross section in *Ambn*^WT/WT^ (c), *Ambn*^ΔL76-P86 +/−^ (f) and *Ambn*^ΔL76-P86 −/−^ (i). White arrowheads in (e, f), represent large empty gaps within the prisms. White arrows in (e, f) represent increase in the interprismatic enamel at the cost of prismatic enamel. Black arrow represents dark grey patches observed at low resolution with no hierarchical mineral organization (h).

The enamel on the labial side of the *Ambn*^WT/WT^ and *Ambn*^ΔL76-P86 +/−^ incisors appeared smooth (Supplementary Figure 3 a, b and d, e), while that of *Ambn*^ΔL76-P86 −/−^ exhibited a rough “sandpaper” like texture with spherical nodules on the surface and significant blunting of incisal tips (Supplementary Figure 3 c, f). Low-resolution cross-sectional BSE images of *Ambn*^WT/WT^ reveals a uniform and highly mineralized enamel layer (Figure 3a). Two regions were identified along the cross-section of incisors which were examined at a higher resolution. At both regions, a tight packing density of crystallites and a regular ordered rod-interrod enamel with evidence of decussation was observed in *Ambn*^WT/WT^ (Figure 3 b, c). Substantial alterations of the enamel ultrastructure were observed in both *Ambn*^ΔL76-P86 +/−^ and *Ambn*^ΔL76-P86 −/−^ mice. In both regions of *Ambn*^ΔL76-P86 +/−^ interrod enamel areas were thicker suggesting an increase in the interrod enamel at the expense of rod enamel (Figure 3 e, f white arrows). Additionally, large empty gaps were observed in place of rod enamel crystallite bundles in *Ambn*^ΔL76-P86 +/−^ (Figure 3 e, f white arrowheads). Cross-sectional BSE of *Ambn*^ΔL76-P86 −/−^ revealed the presence of large grey areas of low mineral density (Figure 3g, black arrow). While some rod-interrod structures were discernable in *Ambn*^ΔL76-P86 +/−^, a lack of prismatic enamel organization was obvious in the *Ambn*^ΔL76-P86 −/−^ in both regions (Figure 3 h, i).

### Ambn-ameloblast cell membrane interactions are lost in the *Ambn*^ΔL76-P86^ mutants

In secretory stage of *Ambn*^WT/WT^ labeling with a custom-made N-terminal Ambn antibody was specific to the Tomes’ process (TPs) and distal ameloblast membrane including the secretory vesicles (Supplementary Figure 4a, white arrows). This labeling pattern persists in the transition stage (Supplementary Figure 4b). In the maturation stage, however, the ECM labeled strongly for the N-terminal fragment, with a characteristic cross-hashing pattern of labeling reminiscent of the Ambn fragments persisting around the prism boundaries in the prism sheath^35^ (Supplementary Figure 4c, white arrows).

**Figure 4.**
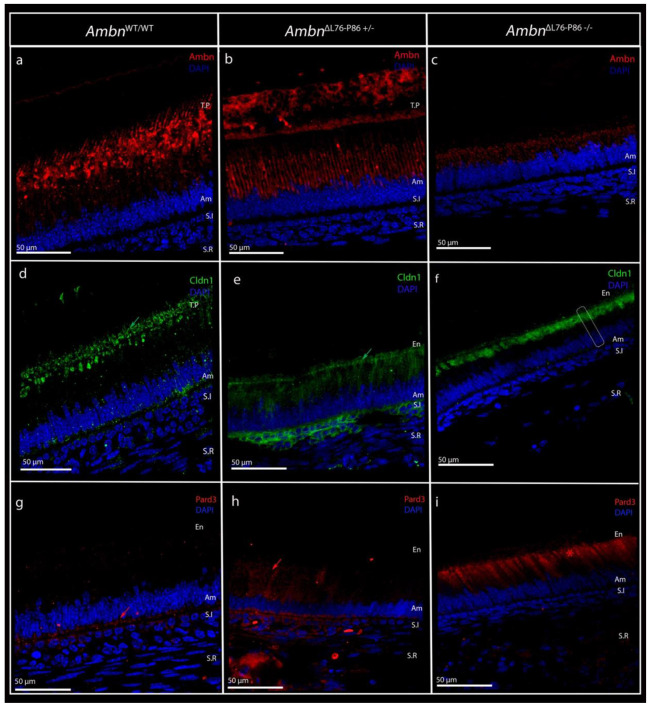
Confocal microscopy images of P8 incisor showing ameloblastin AH motif-ameloblast distal cell membrane interaction (top panel) and ameloblast cell membrane polarity characterization in *Ambn*^WT/WT^ and *Ambn*^ΔL76-P86^ mutants during secretory stage (middle and bottom panels). (**a - c**) Immunolabeling using a custom N-terminal Ambn peptide antibody (red) in *Ambn*^WT/WT^ (a), *Ambn*^ΔL76-P86 +/−^ (b) and *Ambn*^ΔL76-P86 −/−^ (c). Immunosignals for N-terminal Ambn in *Ambn*^WT/WT^ are observed specifically along the distal ameloblast membrane and Tomes’ processes. (**d - f**) Labeling of tight-junctional protein claudin-1, Cldn1 (green), in secretory stage ameloblasts of *Ambn*^WT/WT^ (d), *Ambn*^ΔL76-P86 +/−^ (e), and *Ambn*^ΔL76-P86 −/−^(f). Green arrow in (d) represents Cldn1 immunosignals along the Tomes’ processes and distal membrane and green arrow in (e) represent immunosignal of Cldn1 along the distal terminal web. Dotted lines in (f) used to highlight ameloblast outline. (**g - i**) Immunolabeling of cell polarity protein, Pard3 (red), in the secretory stage ameloblasts of *Ambn*^WT/WT^ (g), *Ambn*^ΔL76-P86 +/−^ (h), and *Ambn*^ΔL76-P86 −/−^(i). Red arrow in (g) used to denote Pard3 signals along the proximal ameloblast membrane and red arrow in (h) used to indicate the increase in cytoplasmic levels of Pard3 in *Ambn*^ΔL76-P86+/−^. Red asterisks in (i) represent mis-localization of Pard3 in the intracellular regions. En- enamel, T.P- Tomes’ processes, Am- ameloblast, S.I stratum intermedium, S.R stellate reticulum.

Using the custom anti-Ambn antibody revealed that in the *Ambn*^WT/WT^ samples, the N-terminal Ambn fragment specifically immunolocalized with the distal ameloblast membrane and along the long slender TPs, thereby resulting in a highly polarized distribution within the ameloblast (Figure 4a). In the *Ambn*^ΔL76-P86 +/−^ a peculiar labeling pattern was present (Figure 4b). Immunosignals were present both within the ameloblasts as well as in the ECM. The intracellular labeling in *Ambn*^ΔL76-P86 +/−^ was confined to the supranuclear region, with no discernable labeling along the distal ameloblast membrane. In the *Ambn*^ΔL76-P86 −/−^ samples, N-terminal Ambn labeling was confined intracellularly within the secretory stage ameloblasts (Figure 4c). The immunosignals for Ambn were restricted to the immediate supranuclear region, with little to no labeling along the distal membrane and Tomes’ processes (Figure 4c).

We confirmed the specific loss of membrane localization in the *Ambn*^ΔL76-P86 −/−^ mutants compared to *Ambn*^WT/WT^ by co-labeling of β-actin and N-terminal anti-Ambn in secretory stage (Supplementary Figure 5). In the *Ambn*^WT/WT^ secretory stage, co-localization between β-actin (green) and N-terminal Ambn (red) was specific to the distal membrane and TPs (Supplementary Figure 5 a - d). In the *Ambn*^ΔL76-P86 −/−^ most of N-terminal Ambn immunosignals (red) were within the cell, and only faint labeling was observed along the distal membrane resulting in little to no colocalization between β-actin (green) and N-terminal Ambn (red) (Supplementary Figure 5 e - h). Overall, removal of LP region in *Ambn*^ΔL76-P86^ mutants resulted in a lack of Ambn immunolocalization to the distal ameloblast cell membrane *in vivo*.

### Ameloblastin-ameloblast distal membrane interaction predicates polarity development *in vivo*

We have recently demonstrated that Ambn directly interacts with ameloblast cell membranes through the AH motif.^28^ Here, we examined the onset of ameloblast cell polarity and Ambn-ameloblast distal cell membrane interaction *in vivo*. Using stagewise co-labeling in *Ambn*^WT/WT^ we observed that the onset of Pard3 polarity in presecretory stage ameloblasts coincides with the establishment of ameloblastin distal membrane interaction (Supplementary Figure 6). In the pre-ameloblast stage, the cells are shorter with the nucleus central in its positioning within the cell (Supplementary Figure 6a). Immunosignals for Pard3 (red) in this stage were diffuse, and that for full-length Ambn (yellow pseudo-color) were intracellular and faint. The onset of polarity in presecretory ameloblasts was characterized by a massive expansion of cytoplasmic volume, resulting in cell elongation and the movement of the nucleus towards the proximal ameloblast membrane (Supplementary Figure 6b). Immunosignals for Pard3 at this stage were highly polarized and restricted to the vicinity of the proximal ameloblast membrane (Supplementary Figure 6b, red arrows), and this coincided with the immunolocalization of Ambn to the distal ameloblast membrane (Supplementary Figure 6b, yellow arrows).The polarized Pard3 distribution within the ameloblasts, and Ambn along the distal ameloblast membrane and TPs continued into the secretory stage (Supplementary Figure 6c).

### *Ambn*^ΔL76-P86^ mutants exhibit loss of ameloblast polarity and blunted Tomes’ processes

**Figure 5.**
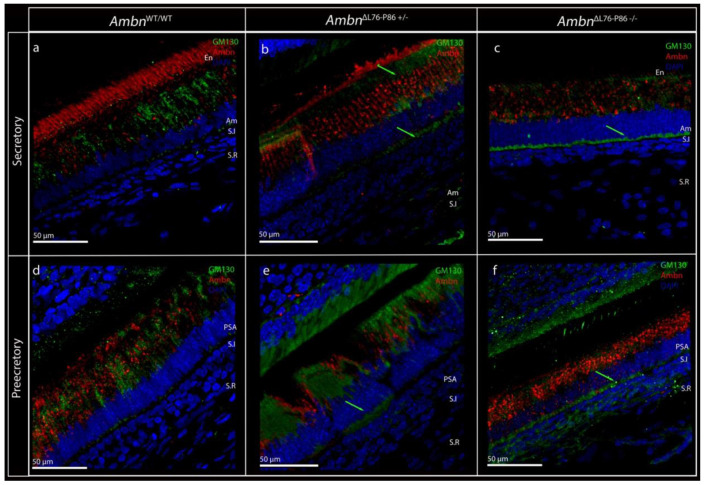
Confocal microscopy images of P8 incisor showing ameloblast polarity in *Ambn*^WT/WT^ and *Ambn*^ΔL76-P86^ mutants examined at the secretory and presecretory stages, at the level of intracellular organelles. (**a - c**) Secretory stage ameloblasts from P8 incisors of *Ambn*^WT/WT^ (a), *Ambn*^ΔL76-P86 +/−^ (b), and *Ambn*^ΔL76-P86 −/−^ (c) labeled with cis-Golgi marker, GM130 (green), and full-length anti-ameloblastin antibody (red). Green arrow in (b) represents GM130 immunosignals at the supra- and infranuclear region in the case of *Ambn*^ΔL76-P86 +/−^ mutant. In the *Ambn*^ΔL76-P86 −/−^ mutant, GM130 signals are observed primarily only in the infra-nuclear region (green arrow in c), in stark contrast with *Ambn*^WT/WT^. (**d - f**) Presecretory stage ameloblasts from P8 incisor in *Ambn*^WT/WT^ (d), *Ambn*^ΔL76-P86 +/−^ (e), and *Ambn*^ΔL76-P86 −/−^ (f). In *Ambn*^WT/WT^ samples, supranuclear signals for GM130 can be observed from the presecretory stage onwards. In the *Ambn*^ΔL76-P86 +/−^ samples, similar to the secretory stage, GM130 signals are observed in both supra- and infranuclear regions (green arrow in e). In the *Ambn*^ΔL76-P86 −/−^, signals are primarily restricted to the infranuclear region of the stunted presecretory stage ameloblasts. En- enamel, Am- ameloblast, S.I stratum intermedium, S.R stellate reticulum, PSA- presecretory ameloblast.

We hypothesized that the lack of membrane interaction in the LP mutant would negatively impact ameloblast polarity development. To test this, ameloblast polarity was examined at the level of the cell membrane (Figure 4) and at the level of intracellular organelles in the mutants compared to the *Ambn*^WT/WT^ animals (Figure 5). Immunolabeling of tight junctional protein claudin-1 (Cldn1) (green), and cell polarity protein, Pard3 (red) was used to characterize ameloblast cell membrane polarity. In fully polarized secretory ameloblasts, cell polarity protein Pard3 and tight junctional protein Cldn1 label along opposite ameloblast membranes.^36^ Consistent with this finding in literature, in *Ambn*^WT/WT^ secretory stage, Cldn-1 labeling was observed along the TPs in the distal ameloblast membrane (Figure 4d, green arrow). In the secretory stage ameloblasts of *Ambn*^ΔL76-P86 +/−^, Cldn-1 labeling was observed primarily in the distal terminal web (Figure 4e, green arrow) with some labeling along the short TPs. Remarkably, this labeling pattern was lost in the *Ambn*^ΔL76-P86 −/−^ mutant. Cldn1 was sequestered within the cytoplasm in the vicinity of the distal ameloblast membrane (Figure 4f, dotted lines show ameloblast outline), with no discernable labeling along the distal membrane. Pard3 labeling in *Ambn*^WT/WT^ secretory stage was restricted to the immediate vicinity of the proximal ameloblast membrane (Figure 4g, red arrow). In the *Ambn*^ΔL76-P86 +/−^ mutants, there was an increase in the levels of Pard3 labeling in the cytoplasm compared to *Ambn*^WT/WT^ (Figure 4h, red arrow). In the *Ambn*^ΔL76-P86 −/−^ samples, we observed a sequestration of Pard3 immunosignals within the cytoplasm, similar to observations with Cldn1 labeling (Figure 4i, red asterixis).

Golgi labeling (using GM130 as a marker) in the *Ambn*^WT/WT^ secretory stage ameloblasts was restricted primarily to the supranuclear region of the cells, with only minimal labeling along the infranuclear region (Figure 5a). This distinct labeling pattern was present right from the presecretory stage (Figure 5d), which corresponded to the onset of ameloblast polarity characterized by Golgi movement around the nucleus.^8^ Although a majority of the immunosignals for GM130 in *Ambn*^ΔL76-P86 +/−^ were in the supranuclear compartment of the secretory and presecretory stage ameloblasts, the intensity of labeling in the infranuclear region was greater than that of *Ambn*^WT/WT^ in both the presecretory and secretory stages (Figure 5 b, e green arrows). In *Ambn*^ΔL76-P86 −/−^ samples we observed a strong and distinctly different labeling that was primarily restricted to the infranuclear region, between the nucleus and the proximal ameloblast membrane in the secretory stage (Figure 5c, green arrow). The prominent supranuclear labeling of GM130 in the presecretory stage of amelogenesis in *Ambn*^WT/WT^ was not observed in presecretory stage ameloblasts of *Ambn*^ΔL76-P86 −/−^ (Figure 5f).

### Defective ameloblast polarity in *Ambn*^ΔL76-P86^ mutants impacts ameloblast elongation

**Figure 6.**
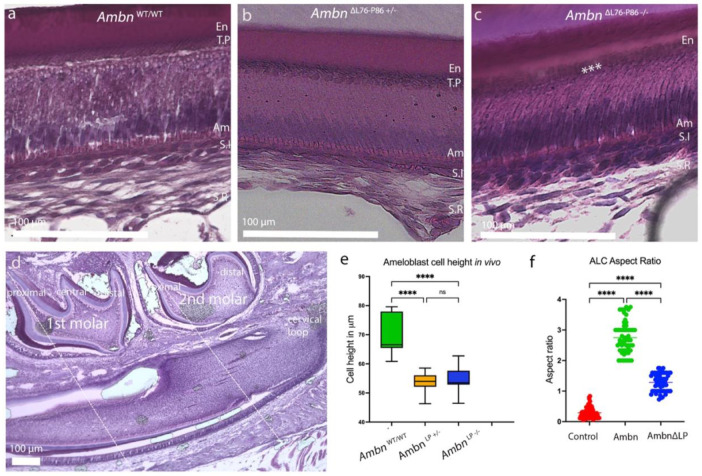
Effect of loss of polarity in *Ambn*^ΔL76-P86^ mutation on ameloblast morphology and aspect ratio *in vivo* and *in vitro*. (**a - c**) High magnification histological H&E staining images of P8 incisors taken from *Ambn*^WT/WT^ (a), *Ambn*^ΔL76-P86 +/−^ (b) and *Ambn*^ΔL76-P86 −/−^(c). Ameloblast height in *Ambn*^ΔL76-P86 +/−^ and *Ambn*^ΔL76-P86 −/−^ appear stunted in comparison to *Ambn*^WT/WT^. (**d**) Low magnification image of P8 *Ambn*^WT/WT^ incisor H&E staining showing the anatomical landmarks used to identify ameloblast stages. The proximal cusps of 1^st^ and 2^nd^ molars are used as the guide to locate secretory and presecretory ameloblast respectively as indicated by dotted white lines in d. (**e**) In vivo secretory ameloblast height measurement from the H&E-stained images. *Ambn*^WT/WT^ (green), *Ambn*^ΔL76-P86 +/−^ (yellow) and *Ambn*^ΔL76-P86 −/−^ (blue). (**f**) Ameloblast lineage cell (ALC) aspect ratio changes as the result of ameloblastin addition to a 3D cell culture as measured by a ratio of ameloblast cell height to width. Heat denatured recombinant mouse ameloblastin control (red), recombinant mouse Ambn (green) and recombinant mouse AmbnΔL76-P86 (blue). En- enamel, T.P-Tomes’ process, Am- ameloblast, S.I stratum intermedium, S.R stellate reticulum. * *p* < 0.05; **p < 0.01, *** *p* < 0.001, n/s- not significant.

We examined the effect of loss of polarity in the *Ambn*^ΔL76-P86^ mutants on amelogenesis by evaluating its effects on ameloblast morphology (elongation) *in vivo* and *in vitro*. Secretory ameloblasts in *Ambn*^WT/WT^ revealed a characteristic elongated, polarized columnar morphology with distal TPs (Figure 6a). In the case of *Ambn*^ΔL76-P86 +/−^, ameloblasts appeared shortened and TPs were less defined as compared to *Ambn*^WT/WT^ (Figure 6b). In the *Ambn*^ΔL76-P86 −/−^ secretory stage ameloblasts appeared stunted with an obvious reduction in their height (Figure 6c). The overall nuclear to cytoplasmic ratio was altered in the ameloblasts of *Ambn*^ΔL76-P86 −/−^.

Additionally, TPs were not discernable (Figure 6c, asterisks). We used the 1^st^ and 2^nd^ mandibular molar proximal cusps along with the cervical loop as anatomical landmarks to identify the secretory and presecretory stages of amelogenesis within the continuously growing mouse incisors (Figure 6d) (Supplementary Figure 7) ^37^. Measurements of secretory ameloblast height from *Ambn*^WT/WT^ revealed an average height of 65.22 μm (n=5) (Figure 6e). In the *Ambn*^ΔL76-P86 −/−^ secretory ameloblasts were significantly shorter, measuring only 56.26 μm (*p* < 0.001) (n=5) (Figure 6e). There was no significant difference in the secretory ameloblast height between *Ambn*^ΔL76-P86 +/−^ and *Ambn*^ΔL76-P86 −/−^ (Figure 6e), nor in the presecretory ameloblast height (Supplementary Figure 7).

Using a recently developed *in vitro* model of 3D-on-top type culture,^29^ we observed that ALC cultured in the presence of WT recombinant mouse Ambn elongated preferentially along the +Z axis. As a result, the aspect ratio of cells (height/ width) was 2.75 +/− 0.18 which was significantly greater than the negative control (heat denatured Ambn) (Figure 6f). ALC elongation in the presence of recombinant mutant AmbnΔL76-P86 was significantly lower compared WT recombinant Ambn (*p* < 0.0001) (Figure 6f).

### Proximal adherens junction stability and β-catenin localization are altered in *Ambn*^ΔL76-P86^ mutants

**Figure 7.**
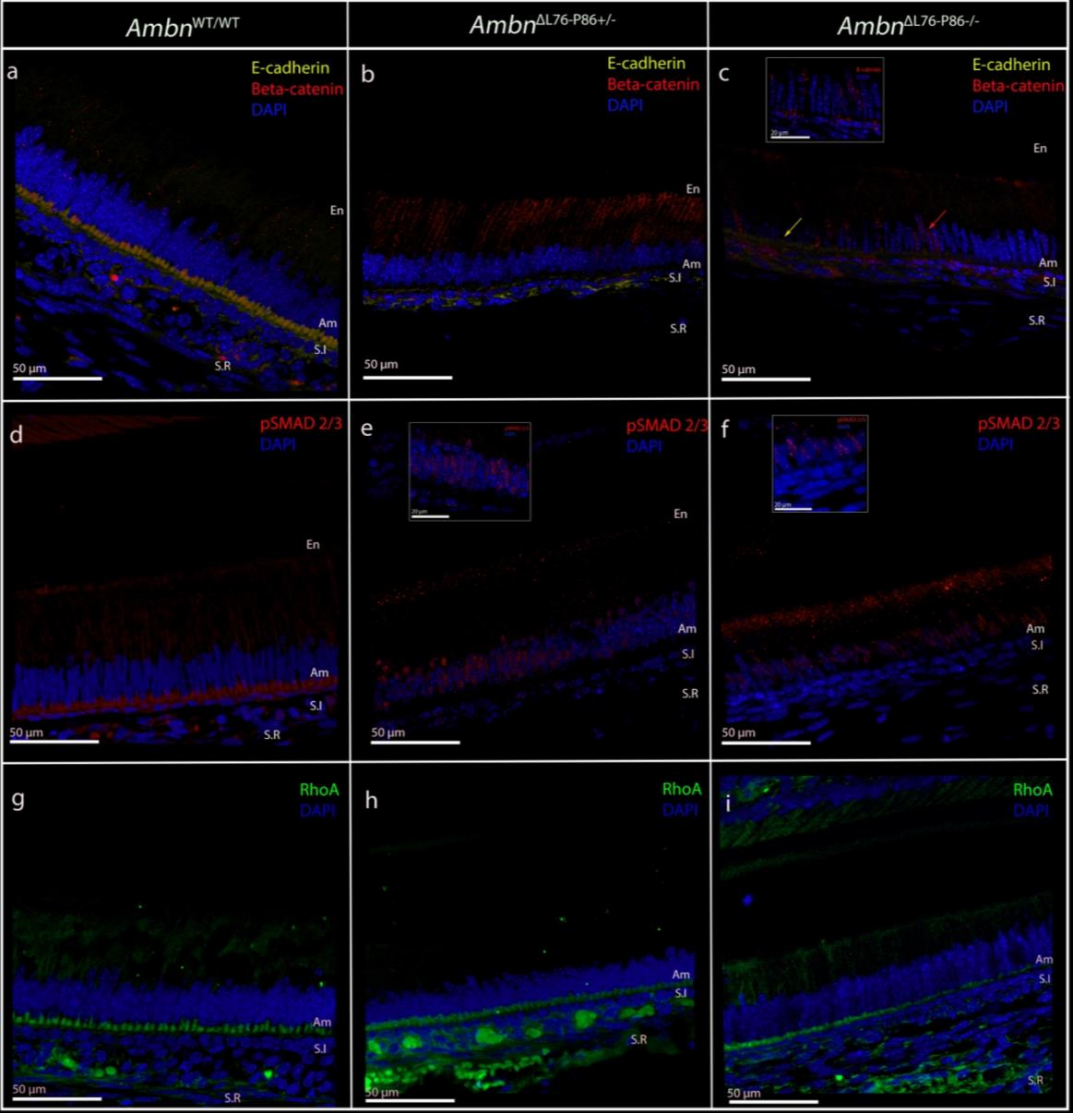
Confocal microscopy images of P8 incisor showing expression of E-cadherin, β-catenin, p-Smad 2/3, and RhoA as indication of cell polarity mechanisms and associated signaling pathways in *Ambn*^WT/WT^ and *Ambn*^ΔL76-P86^ mutants. (**a - c**) Immunolabeling for E-cadherin (yellow) and β-catenin (red) in secretory stage ameloblasts from *Ambn*^WT/WT^ (a), *Ambn*^ΔL76-P86+/−^ (b), and *Ambn*^ΔL76-P86 −/−^ (c). In the case of *Ambn*^ΔL76-P86 −/−^ there is an increase in the nuclear localization of β-catenin that is observed (red arrow in c and inset) with a concomitant decrease in the immunolabeling intensity for E-cadherin (yellow arrow, c). (**d - f**) Immunolabeling for p-Smad 2/3 in *Ambn*^WT/WT^ (d), *Ambn*^ΔL76-P86 +/−^ (e), and *Ambn*^ΔL76-P86 −/−^ (f). Immunosignals for p-Smad 2/3 in *Ambn*^WT/WT^ is in the cytoplasm of the ameloblasts. In the case of the *Ambn*^ΔL76-P86^ mutants, an increase in the nuclear localization of p-Smad 2/3 is observed (e, f inset) suggesting potential alterations in the canonical TGF-β signaling pathway. (**g - i**). Immunolabeling for RhoA in *Ambn*^WT/WT^ (g), *Ambn*^ΔL76-P86 +/−^ (h), and *Ambn*^ΔL76-P86 −/−^ (i). RhoA immunosignals are present in *Ambn*^ΔL76-P86^ mutants at a comparable localization to *Ambn*^WT/WT^. However, signal intensity is lower in the *Ambn*^ΔL76-P86−/−^ mutants. En- enamel, Am- ameloblast, S.I stratum intermedium, S.R stellate reticulum.

We examined and characterized the molecular mechanisms underlying the lack of development of a fully polarized ameloblast in the *Ambn*^ΔL76-P86^ mutants by immunolabeling for E-cadherin and β-catenin and compared with *Ambn*^WT/WT^. Co-labeling with E-cadherin (yellow) and β-catenin (red) in *Ambn*^WT/WT^ secretory stage ameloblasts reveals their near complete colocalization along the proximal ameloblast membrane (Figure 7a). In the case of *Ambn*^ΔL76-P86 +/−^, immunosignals for β-catenin were localized primarily to the cytoplasm of the ameloblasts resulting in a decreased immunolabeling intensity for E-cadherin along the proximal ameloblast membrane (Figure 7b). In the *Ambn*^ΔL76-P86 −/−^ secretory stage ameloblasts, β-catenin was not found to colocalize with E-cadherin and was instead translocated to the nuclear compartment (Figure 7c red arrows and inset).

### Dysregulated TGF-β1 signaling in *Ambn*^ΔL76-P86^ mutants

To gain insight into possible signaling pathways involved we examined both the canonical TGF-β1 pathway and the MAPK pathways by labeling for p-Smad 2/3 and p-Erk1/2 respectively. Labeling for p-Smad 2/3 in the *Ambn*^WT/WT^ revealed positive immunolabeling in the cytoplasm of the secretory stage ameloblast, consistent with literature (Figure 7d). ^38^ We observed an increase in the nuclear translocation of p-Smad 2/3 in the case of both *Ambn*^ΔL76-P86 +/−^ (Figure 7e, inset) and *Ambn*^ΔL76-P86 −/−^ (Figure 7f, inset). Labeling for p-Erk1/2 in the secretory stage ameloblasts of *Ambn*^WT/WT^ was negative and there was no change in the p-Erk1/2 labeling in the *Ambn*^ΔL76-P86^ mutants compared to *Ambn*^WT/WT^ (Supplementary Figure 8).

### A decrease in the secretory stage RhoA signal intensity in the *Ambn*^ΔL76-P86^ mutants

RhoA immunosignals in the *Ambn*^WT/WT^ and *Ambn*^ΔL76-P86^ mutants were primarily present along the proximal ameloblast cell membrane (Figure 7 g - i). We did not observe any changes in the localization of RhoA immunosignals in the *Ambn*^ΔL76-P86^ mutants. However, the signal intensity appeared reduced in the *Ambn*^ΔL76-P86 −/−^ mutants. This was confirmed using measurements of RhoA signal intensity at the proximal membrane. Normalized RhoA signal intensity comparison revealed a significant reduction in the RhoA intensity in the *Ambn*^ΔL76-P86 −/−^ mutant compared to *Ambn*^WT/WT^ and *Ambn*^ΔL76-P86 +/−^ (*p* < 0.05) (Supplementary Figure 9).

## Discussion

To examine the biological function of the evolutionary conserved AH motif within the multitargeting domain of enamel matrix protein Ambn, we designed a mutation in a manner to selectively disrupt the AH motif. Our objective was to examine the specific effects of loss of a functional AH motif on enamel formation. We hypothesized that disturbances in ameloblastin cell membrane interactions, consequent to AH motif disruption, will affect cell polarization and prismatic mineral organization. We deleted eleven amino acids within the AH motif that are primarily hydrophobic in nature (called LP region). We used recombinant proteins and peptides with similar deletion in *in vitro* and cell culture experiments to confirm disturbance to the cell binding function. The *in vitro* experiments supported the design of our animal models and confirmed that the cell binding AH motif is specifically targeted without significantly affecting the self-assembly properties of the protein.

Loss of a functional AH motif in the *Ambn*^ΔL76-P86^ mouse mutants resulted in the formation of enamel that lacked prismatic structure and had a significantly lower mineral density but normal thickness. Spatial metrics along the arc of the incisors showed that the initiation of secretory stage in the mutants was delayed. Ameloblasts from the *Ambn*^ΔL76-P86^ mutants did not show any overt signs of pathology, except for a stunted morphology with defective cell polarization (Golgi positioning as well as cell membrane domains). A mouse model with disruptions of the highly conserved self-assembly (Y/F-x-x-Y/L/F-x-Y/F) motif using an amino acid substitution model (*Ambn*^G/G^) presents a completely different phenotype.^19^ The disruption of the self-assembly motif without concomitant changes in the AH motif in the *Ambn*^G/G^ model shows no changes in ameloblast morphology or polarization. These observations collectively suggest the Ambn multitargeting region contains domains with distinct functionality.

The normal enamel thickness observed in our mutant animals is in contrast with previous *Ambn* mutant animal models.^21-23^ Importantly, RNA sequencing demonstrates that the expression levels of *AmelX* and *Enam* genes are normal in our mutants. Evolutionary analysis and experimental evidence support the notion that amelogenin protein is necessary for the growth of elongated hydroxyapatite crystals, and for the expansion of enamel matrix thickness. ^39-41^ Enamelin (*ENAM*) is the ancestral gene from which *AMBN* and *AMELX* genes arose, and the protein enamelin has been implicated in controlling enamel apatite crystal nucleation. ^42-45^ Collectively, the normal expression levels of the other major EMPs, specifically amelogenin and enamelin, may in part compensate for certain aspects of enamel formation including the elaboration of normal enamel thickness and initiation of mineral nucleation. However, the specific function of AH motif in maintaining ameloblast morphology and polarization could not be compensated as none of the other EMPs have the AH motif.^28^

Using co-labeling of a custom anti-Ambn antibody with β-actin, we demonstrated a loss of Ambn-ameloblast distal cell membrane interactions in the *Ambn*^ΔL76-P86^ mutants. We postulated that the lack of membrane interaction would inhibit the development of ameloblast polarity. To test this, we first co-labeled Ambn and cell polarity protein Pard3 in developing ameloblasts from mice. In the case of wildtype ameloblasts, Pard3 labeling was shown to be specific to the proximal ameloblast cell membrane in association with the proximal tight junctions.^36^ Using systematic labeling in the presecretory ameloblasts, we show that the onset of Pard3 polarity (as shown by its asymmetrical labeling within the ameloblast proximal membrane) coincided with the immunolocalization of Ambn to the distal ameloblast cell membrane. Our data corroborate with recent reports that recombinant mouse Ambn induced an increase in the expression levels of *Pard3* in an ameloblast cell lines^31^ and resulted in polarization immunolocalization of Pard3 within the clusters of elongated cells in 3D cultures.^29,30^

**Scheme 1.**
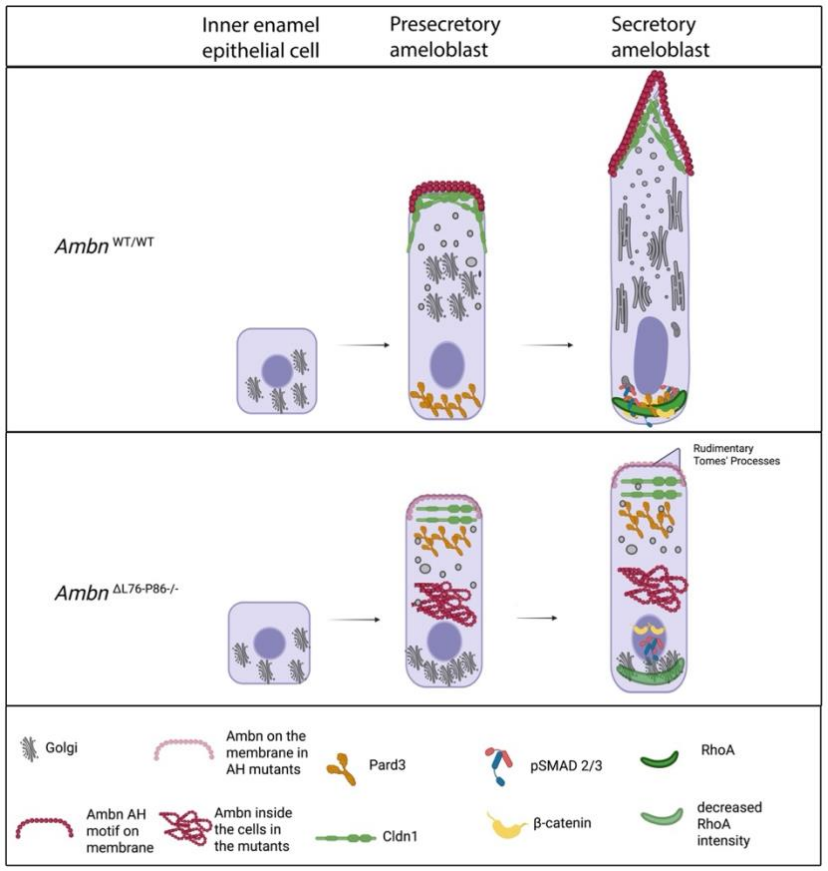
Schematic representation summarizing Ambn-cell membrane interactions and the expression of polarization markers in *Ambn*^WT/WT^ (top) and *Ambn*^ΔL76-P86^ mutants (bottom).

The observations at the level of the intracellular organelles as well as the cell membrane in our AH mutant animals showed a significant lack of development of cell polarity in the homozygou*s Ambn*^ΔL76-P86^ mutant compared to *Ambn*^WT/WT^. In contrast, in the heterozygous *Ambn*^ΔL76-P86^ mutant, cell membrane and intracellular organelle polarity was affected to a lesser extent. Tight junctional protein *Cldn-1* transcripts have been shown to be upregulated in the highly elongated presecretory stage ameloblasts compared to the shorter inner enamel epithelial cells.^46^ Cldn-1 localization in secretory ameloblasts is primarily along the distal membrane and TPs. ^36,46^ (Scheme 1). Removal of the LP region resulted in an increase in the cytoplasmic levels of Cldn-1 with little to no membrane localization, resulting in a potential loss of distal tight junction assembly. Tight junctions (TJs) are crucial for paracellular permeability, and for restricting the intermixing of apical and basolateral membrane domains. ^47^ Additionally, components of the Crumbs complex homolog, Crb3 have been shown to interact with Cldn-1. ^48^ In secretory stage ameloblasts the proximal TJs have been shown to be structurally leaky while the distal TJs exhibit a more robust structure that likely function to prevent the movement of calcium ions. ^49,50^ These observations collectively highlight that the sequestration of Cldn-1 in the cytoplasm of the mutants likely affects the permeability barrier function as well as the correct positioning of membrane domains in the mutant ameloblasts.

The ability of epithelial cells to re-arrange their membrane domains in response to changes in the ECM is well known and characterized.^51-53^ A unique characteristic that distinguishes ameloblasts is the reversal of polarity during the early stage of enamel development. Ameloblast reversal of polarity has been defined based on electron microscopy observations of the Golgi positioning. ^1,12^ Using GM130 immunolabeling, we observed that the movement of Golgi around the nucleus was impaired in the case of the *Ambn*^ΔL76-P86^ mutants, beginning from the presecretory stage. Ameloblast elongation was also impaired in the *Ambn*^ΔL76-P86^ mutant animals. Notably, *Ambn*^ΔL76-P86^ mutant cells were 19-23% shorter than those of *Ambn*^WT/WT^. Recombinant Ambn induced the selective elongation of ameloblast cell lines along the +Z axis in the independent 3D culture experiments.^29,30^ Here we show that disruption of the AH motif resulted in a significant impairment of this function both *in vitro* and *in vivo*. Given that secretion of EMPs occurs towards the former anatomical base in ameloblasts, we suggest that the exaggerated elongation in the secretory stages might function to accentuate the asymmetry within the cells and aid in protein secretion.

Several Wnt pathway genes have been implicated in tooth development ^54,55^ , and it has been shown that constitutive activation of β-catenin in ameloblasts results in enamel hypomineralization defects ^56^. Treatment of ameloblasts with GSK3β inhibitor results in ameloblast polarity defects due to an enhancement of Wnt signaling ^57^. In both homozygous and heterozygous the *Ambn*^ΔL76-P86^ mutants, the integrity of the ameloblast proximal adherens junction was compromised with potential changes in Wnt signaling secondary to the nuclear translocation of β-catenin ^58^. A hyperactive TGF-β1 signaling in amelogenesis has been shown to result in enamel mineralization defects in genetically engineered models of Smad2 overexpression ^59^. Additionally, a hyperactive MAPK pathway (characterized by an increase in the p-Erk 1/2 levels) has been shown to drive the pathogenesis of ameloblast polarity defects in mouse models of Costello syndrome ^60^. Notably, we did not observe any changes in the p-Erk levels in the *Ambn*^ΔL76-P86^ mutants; however, an increase in the nuclear localization of p-Smad 2/3 suggests potential alterations in the canonical TGF-β1 pathway.

Both RhoA as well as its downstream effector Rock have been extensively characterized in ameloblasts ^61-64^. Active RhoA immunosignals are upregulated in the polarized ameloblasts compared to the shorter cuboidal pre-ameloblasts ^65^. RhoA in ameloblasts has also been shown to be necessary for the correct polarized cortical distribution of E-cadherin ^65^ . Both Rock isoforms are expressed in ameloblasts ^63^. Treatment of ameloblasts with Rock inhibitor Y27632 resulted in a loss of polarized distribution of E-cadherin and β-catenin as well as disruptions to the directional secretion of Amel and Ambn ^63^. We have previously shown that treatment of ameloblast cell lines with recombinant Ambn resulted in an upregulation of *Rock1* and *Rock2* isoforms when compared to denatured Ambn ^31^. Taken together with the observations regarding a decrease in the RhoA signal intensity in *Ambn*^ΔL76-P86 −/−^ secretory stage ameloblasts, we suggest that AH motif likely functions through RhoA signaling pathway.

In summary, we propose that interaction of Ambn with the ameloblast membrane via the AH motif specifies a unique apical membrane identity and participates in cell signaling interactions that function to establish a prismatic enamel structure through control of ameloblast morphology and polarization. The animal model presented here enables the elucidation of molecular mechanisms underlying the pathogenesis of human hereditary enamel defects involving Ambn AH motif. Heterogenous phenotypic presentations in patients with *Amelogenesis Imperfecta* involving a truncation of the AH motif, further illustrate the dynamic multifunctionality of this region ^16,24^. Additionally, the newly identified signaling functions of the Ambn AH motif indicate a potential application for use in tissue engineering.

## MATERIALS AND METHODS

### Recombinant AH mutant protein design, expression and purification

An ameloblastin mutant was designed to delete the hydrophobic residues within the evolutionarily conserved AH motif, AmbnΔL76-P86. The details of the expression construct, and recombinant protein expression and purification are elaborated in Supplementary Information.

### Generation of ameloblastin AH motif deletion mice

Genetically engineered mice with a disruption of the amphipathic helix (AH) motif through deletion of hydrophobic residues (*Ambn*^ΔL76-P86^) in a C57BL/6 background were generated at the Mouse Biology Program, UC Davis (CA, USA) using CRISPR-Cas9 technology. Heterozygous mice were transferred to the Department of Animal Resources, University of Southern California. Animals were handled as per approved protocols from the Institutional Animal Care and Use Committee (IACUC), University of Southern California. The *Ambn*^ΔL76-P86 +/−^ mice were bred to generate *Ambn*^ΔL76-P86 −/−^ mice which were used in the following experiments. Generation of homozygous mutants was confirmed using agarose gel electrophoresis. The mutant animals were maintained on a soft gel-diet that was nutritionally balanced. Experimental replicates were performed using animals from different breeding cages (non-littermates). n=5 for conventional micro-CT analysis, n=4 for high resolution micro-CT analysis and n=3 for immunofluorescence experiments.

### Micro-CT scanning

Seven-week-old mouse mandibles were dissected and the soft tissues removed using a scalpel. Hemi-mandibles were then scanned using SkyScan 1174 (Bruker) operating at 50 kV, 800 μA, with a resolution of 14.1 μm. A 0.25 mm aluminum filter was used to selectively remove low-energy X-rays and improve image quality. Three-dimensional reconstructions of the raw scans were generated using NRecon (version 1.6.9.8). The reconstructed dataset was exported to DicomCT and visualized using Amira. Detailed protocols for enamel mineral density and thickness measurements from conventional micro-CT scanning, and details of high-resolution micro-CT scanning and analysis are outlined in the Supplementary Information.

### Scanning electron microscopy

Mandibles from seven-week-old mice were dissected, and the soft tissues were removed. The hemi-mandibles were dehydrated using an increasing ethanol gradient and embedded in epoxy resin (Pelco 2-hour Epoxy Mount Kit, Ted Pella, Redding, CA, USA). The embedded hemi-mandibles were sectioned at 1 mm increments from the first molar to the incisor tip. The cross-sections were ground through a series of ascending grits of silicon carbide papers (3M Wetordry Abrasive Sheet) and were polished using a 0.05 μm diamond paste (Buehler). The polished sections were etched with 37% phosphoric acid for 20 s, air-dried, and sputter-coated with a platinum-palladium mixture for 30 s. Samples were examined using scanning electron microscopy (SEM) (Nova NanoSEM 450, FEI, OR, USA) at 10 kV.

### Histology and ameloblast height measurements

Post-natal 8-day old (P8) samples were dissected and fixed (4% paraformaldehyde) overnight and decalcified for 10 days (in 10% EDTA with 0.1% paraformaldehyde). The decalcified mandibles were paraffin embedded and sectioned to obtain 7 μm thick sagittal sections that were stained using hematoxylin and eosin as per standard protocols. First and 2^nd^ mandibular molars, along with the cervical loop were used as anatomical landmarks to orient and identify stages of amelogenesis along the continuously erupting incisors. Presecretory and secretory ameloblast histology was examined and the ameloblast cell height was measured and compared between *Ambn*^ΔL76-P86^ mutants and *Ambn*^WT/WT^.

### Immunofluorescent labeling of P8 incisors and confocal scanning microscopy

P8 mandibular incisors were used for all the immunolabeling experiments. Paraffin sections of 7 μm thickness along the sagittal orientation were prepared, to visualize incisor ameloblasts. A custom antibody was designed with residues 95 - 108 of mouse Ambn as the epitope which was used to specifically immunolocalize the N-terminal Ambn fragment. The following primary antibodies were used: goat polyclonal anti-Ambn (AF3026-SP R&D systems), custom rabbit polyclonal -anti Ambn (Thermo), rabbit polyclonal anti-GM130 (R1608-7 Huabio), rabbit polyclonal anti-Cldn1 (28674-1-AP Proteintech), rabbit polyclonal anti-Pard3 (11085-1-AP Proteintech), mouse monoclonal anti-E-cadherin 555 conjugated (560064, BD Biosciences), rabbit monoclonal anti-β catenin (ab32572 Abcam), rabbit monoclonal anti-pSmad 2/3 (ab202445 Abcam), rabbit monoclonal anti-pErk1/2 (4370T Cell Signaling Technology), mouse monoclonal anti-total RhoA (sc-418 AF488 SCBT, 488 conjugated), mouse monoclonal anti-β actin (ab6277Abcam, 488 conjugated). The following secondary antibodies were used: donkey anti-goat Alexa Fluor 647(705-605-003 Jackson immuno), donkey anti-rabbit Alexa Fluor 647(711-605-152 Jackson Immuno), donkey anti-rabbit Alexa Fluor 488 (A-21206 Thermo), donkey anti-rabbit Alexa Fluor 488 (711-545-152 Jackson Immuno). Detailed protocols for immunofluorescent labeling are outlined in Supplementary Information. The immunolabeled slides were examined using confocal scanning microscopy (Stellaris, Leica) and three-dimensional Z stacks were recorded with an optical pitch of 0.1-0.3 *μ*m. Reconstructed Z stacks were visualized using maximum intensity projection on LasX (Leica). For measurement of RhoA signal intensity, ten regions of interest along the proximal ameloblast membrane were drawn on maximum intensity projections, and the normalized mean signal intensity was calculated and compared between *Ambn*^WT/WT^ and *Ambn*^ΔL76-P86^ mutants.

### Bulk RNA sequencing

Mandibular 1^st^ molar enamel organs were isolated by microscopic dissection from P5 heterozygous and homozygous *Ambn*^ΔL76-P86^ mutants as well as *Ambn*^WT/WT^ mice (2 enamel organs per mouse). Detailed protocols are outlined in Supplementary Information.

### Cell Culture- 3D and 2D

Ameloblast lineage cells (ALC) were obtained from Prof. Toshihiro Sugiyama (University of Akita, Japan (*76*). ALC were cultured in low-glucose Dulbecco’s modified Eagle’s Medium (DMEM) (Corning) supplemented with 10% v/v heat inactivated fetal bovine serum (Gibco) and 100 U/L penicillin and 100 mg/ ml streptomycin. Cells were maintained at 37° in a 5.0% CO_2_ atmosphere. For direct spreading and cell spreading competition assays, established protocols were used ^66^. Detailed methods are outlined in Supplementary Information. ALC were cultured in 3D following published protocols for 3D-on-top type culture ^29,67^. Detailed methods are outlined in the Supplementary Information. All cell culture experiments were carried out in triplicates and repeated thrice.

### Statistical Analysis

Statistical analyses were carried out using three independent experimental repeats using GraphPad Prism (version 10.4.1). The limit for significance was set to *p* < 0.05. All experiments were repeated thrice. Both parametric (One-Way ANOVA) and non-parametric (Kruskal Wallis) tests were used based on the distribution of the datasets. Post hoc tests used were Dunn’s multiple comparison or Sidak’s multiple comparison.

## Supplementary Material

This is a list of supplementary files associated with this preprint. Click to download.

• SupplementaryInformation.pdf

## Data Availability

All data needed to evaluate the conclusions in the paper are present in the paper and/or in the Supplementary Materials. The conventional micro-CT scans have been deposited to FaceBase Consortium.

## References

[1] Warshawsky H. Ultrastructural studies on amelogenesis. The chemistry and biology of mineralized tissues, 33–45 (1985).

[2] Thesleff I. Epithelial-mesenchymal signalling regulating tooth morphogenesis. J. Cell Sci. 116, 1647–1648 (2003). 12665545 10.1242/jcs.00410

[3] Mathur A. K. & Polly P. D. The Evolution of Enamel Microstructure: How Important Is Amelogenin? J. Mamm. Evol. 7, 23–42 (2000). 10.1023/A:1009413632741

[4] Al-Hashimi N., Lafont A.-G., Delgado S., Kawasaki K. & Sire J.-Y. The Enamelin Genes in Lizard, Crocodile, and Frog and the Pseudogene in the Chicken Provide New Insights on Enamelin Evolution in Tetrapods. Molecular Biology and Evolution 27, 2078–2094 (2010). 10.1093/molbev/msq098 20403965

[5] Boyde A. (SAGE Publications, 1967).

[6] Boyde A. in Ciba Found. Symp. 18–31 (Wiley Online Library).

[7] Wood C., Dumont E. & Crompton A. New studies of enamel microstructure in Mesozoic mammals: a review of enamel prisms as a mammalian synapomorphy. J. Mamm. Evol. 6, 177–213 (1999).

[8] Smith C. E. & Nanci A. Overview of morphological changes in enamel organ cells associated with major events in amelogenesis. Int. J. Dev. Biol. 39, 153–161 (2003). 7626402

[9] Matsuo S., Ichikawa H., Wakisaka S. & Akai M. Changes of cytochemical properties in the Golgi apparatus during in vivo differentation of the ameloblast in developing rat molar tooth germs. The Anatomical Record 234, 469–478 (1992). 1456450 10.1002/ar.1092340403

[10] Matsuo S. Changes of lectin staining pattern of the Golgi stack during differentiation of the ameloblast in developing rat molar tooth germs. The Anatomical Record 236, 355–365 (1993). 8338238 10.1002/ar.1092360209

[11] Nanci A. Ten Cate's Oral Histology-e-book: development, structure, and function. (Elsevier Health Sciences, 2017).

[12] Warshawsky H., Josephsen K., Thylstrup A. & Fejerskov O. The development of enamel structure in rat incisors as compared to the teeth of monkey and man. The Anatomical Record 200, 371–399 (1981). 6795971 10.1002/ar.1092000402

[13] Risnes S., Septier D., De Periere D. D. & Goldberg M. TEM observations on the ameloblast/enamel interface in the rat incisor. Connect. Tissue Res. 43, 496–504 (2002). 12489204 10.1080/03008200290000899

[14] Risnes S. The prism pattern of rat molar enamel: a scanning electron microscope study. Am. J. Anat. 155, 245–257 (1979). 474447 10.1002/aja.1001550207

[15] Paine M. L. Regulated gene expression dictates enamel structure and tooth function. Matrix Biol. 20, 273–292 (2001). 11566262 10.1016/s0945-053x(01)00153-6

[16] Kegulian N. C., Visakan G., Bapat R. A. & Moradian-Oldak J. Ameloblastin and its multifunctionality in amelogenesis: A review. Matrix Biol. 131, 62–76 (2024). 38815936 10.1016/j.matbio.2024.05.007 PMC11218920

[17] Su J., Kegulian N. C., Bapat R. A. & Moradian-Oldak J. Ameloblastin binds to phospholipid bilayers via a helix-forming motif within the sequence encoded by exon 5. ACS omega 4, 4405–4416 (2019). 30873509 10.1021/acsomega.8b03582 PMC6410667

[18] Kegulian N. C., Langen R. & Moradian-Oldak J. The Dynamic Interactions of a Multitargeting Domain in Ameloblastin Protein with Amelogenin and Membrane. Int. J. Mol. Sci. 24, 3484 (2023). 36834897 10.3390/ijms24043484 PMC9966149

[19] Wald T. Intrinsically disordered proteins drive enamel formation via an evolutionarily conserved self-assembly motif. Proceedings of the National Academy of Sciences 114, E1641–E1650 (2017). 10.1073/pnas.1615334114 PMC5338493 28196895

[20] Bapat R. A., Su J. & Moradian-Oldak J. Co-immunoprecipitation reveals interactions between amelogenin and ameloblastin via their self-assembly domains. Front. Physiol. 11 (2020). 10.3389/fphys.2020.622086 PMC7786100 33424645

[21] Fukumoto S. Ameloblastin is a cell adhesion molecule required for maintaining the differentiation state of ameloblasts. The Journal of cell biology 167, 973–983 (2004). 15583034 10.1083/jcb.200409077 PMC2172447

[22] Wazen R. M., Moffatt P., Zalzal S. F., Yamada Y. & Nanci A. A mouse model expressing a truncated form of ameloblastin exhibits dental and junctional epithelium defects. Matrix Biol. 28, 292–303 (2009). 19375505 10.1016/j.matbio.2009.04.004 PMC2727877

[23] Liang T. AMBN mutations causing hypoplastic amelogenesis imperfecta and Ambn knockout-NLS-lacZ knockin mice exhibiting failed amelogenesis and Ambn tissue-specificity. Molecular genetics & genomic medicine 7, e929 (2019). 31402633 10.1002/mgg3.929 PMC6732285

[24] Hany U. Novel Ameloblastin Variants, Contrasting Amelogenesis Imperfecta Phenotypes. J. Dent. Res. 103, 22–30 (2024). 38058155 10.1177/00220345231203694 PMC10734210

[25] Delsuc F., Gasse B. & Sire J.-Y. Evolutionary analysis of selective constraints identifies ameloblastin (AMBN) as a potential candidate for amelogenesis imperfecta. BMC Evol. Biol. 15, 148 (2015). 26223266 10.1186/s12862-015-0431-0 PMC4518657

[26] Giménez-Andrés M., Čopič A. & Antonny B. The many faces of amphipathic helices. Biomolecules 8, 45 (2018). 29976879 10.3390/biom8030045 PMC6164224

[27] Drin G. & Antonny B. Amphipathic helices and membrane curvature. FEBS Lett. 584, 1840–1847 (2010). 19837069 10.1016/j.febslet.2009.10.022

[28] Su J., Bapat R., Visakan G. & Moradian-Oldak J. An Evolutionarily Conserved Helix Mediates Ameloblastin-Cell Interaction. J. Dent. Res., 0022034520918521 (2020). 10.1177/0022034520918521 PMC7375739 32401578

[29] Visakan G., Su J. & Moradian-Oldak J. Ameloblastin promotes polarization of ameloblast cell lines in a 3-D cell culture system. Matrix Biol. 105, 72–86 (2022). 34813898 10.1016/j.matbio.2021.11.002 PMC8955733

[30] Visakan G., Bapat R. A., Su J. & Moradian-Oldak J. Modeling ameloblast-matrix interactions using 3D cell culture. Front. Physiol. 13, 1069519 (2022). 36531170 10.3389/fphys.2022.1069519 PMC9751369

[31] Su J., Bapat R. A., Visakan G. & Moradian-Oldak J. Coemergence of the Amphipathic Helix on Ameloblastin With Mammalian Prismatic Enamel. Molecular Biology and Evolution 39, msac205 (2022). 36161489 10.1093/molbev/msac205 PMC9631975

[32] Risnes S. & Li C. Aspects of the final phase of enamel formation as evidenced by observations of superficial enamel of human third molars using scanning electron microscopy. Arch. Oral Biol. 86, 72–79 (2018). 29190456 10.1016/j.archoralbio.2017.11.008

[33] Robinson C., Kirkham J. & Shore R. C. Dental enamel formation to destruction. (CRC press, 2017).

[34] Kegulian N. C. & Moradian-Oldak J. Deletion within ameloblastin multitargeting domain reduces its interaction with artificial cell membrane. J. Struct. Biol. 216, 108143 (2024). 39447937 10.1016/j.jsb.2024.108143 PMC11784912

[35] Uchida T. Immunochemical and immunohistochemical studies, using antisera against porcine 25 kDa amelogenin, 89 kDa enamelin and the 13–17 kDa nonamelogenins, on immature enamel of the pig and rat. Histochemistry 96, 129-138 (1991). 1917569 10.1007/BF00315983

[36] Inai T., Sengoku A., Hirose E., Iida H. & Shibata Y. Differential expression of the tight junction proteins, claudin-1, claudin-4, occludin, ZO-1, and PAR3, in the ameloblasts of rat upper incisors. The Anatomical Record: Advances in Integrative Anatomy and Evolutionary Biology: Advances in Integrative Anatomy and Evolutionary Biology 291, 577–585 (2008). 10.1002/ar.20683 18384062

[37] Smith C. & Nanci A. A method for sampling the stages of amelogenesis on mandibular rat incisors using the molars as a reference for dissection. The Anatomical Record 225, 257–266 (1989). 2683870 10.1002/ar.1092250312

[38] Li S. & Pan Y. Differential expression of transforming growth factor-beta1, connective tissue growth factor, phosphorylated-SMAD2/3 and phosphorylated-ERK1/2 during mouse tooth development. J. Mol. Histol. 48, 347–355 (2017). 28825193 10.1007/s10735-017-9733-4

[39] Slavkin H. C. & Diekwisch T. G. Molecular strategies of tooth enamel formation are highly conserved during vertebrate evolution. Dental Enamel 1997, 73–84 (1997). 10.1002/9780470515303.ch6 9189618

[40] Satchell P. G. Conservation and variation in enamel protein distribution during vertebrate tooth development. J. Exp. Zool. 294, 91–106 (2002). 12210110 10.1002/jez.10148

[41] Gibson C. W. Amelogenin-deficient mice display an amelogenesis imperfecta phenotype. J. Biol. Chem. 276, 31871–31875 (2001). 11406633 10.1074/jbc.M104624200

[42] Deutsch D., Catalano-Sherman J., Dafni L., David S. & Palmon A. Enamel matrix proteins and ameloblast biology. Connect. Tissue Res. 32, 97–107 (1995). 7554940 10.3109/03008209509013710

[43] Tanabe T., Aoba T., Moreno E., Fukae M. & Shimuzu M. Properties of phosphorylated 32 kd nonamelogenin proteins isolated from porcine secretory enamel. Calcif. Tissue Int. 46, 205–215 (1990). 2106381 10.1007/BF02555046

[44] Hu J. C.-C. Enamel defects and ameloblast-specific expression in Enam knock-out/lacz knock-in mice. J. Biol. Chem. 283, 10858–10871 (2008). 18252720 10.1074/jbc.M710565200 PMC2447669

[45] Hu J. C.-C. Enamelin is critical for ameloblast integrity and enamel ultrastructure formation. PLoS One 9 (2014). 10.1371/journal.pone.0089303 PMC3945975 24603688

[46] Zhang Y. SATB1 establishes ameloblast cell polarity and regulates directional amelogenin secretion for enamel formation. BMC Biol. 17, 1–16 (2019). 31830989 10.1186/s12915-019-0722-9 PMC6909472

[47] Zihni C., Mills C., Matter K. & Balda M. S. Tight junctions: from simple barriers to multifunctional molecular gates. Nature reviews Molecular cell biology 17, 564–580 (2016). 27353478 10.1038/nrm.2016.80

[48] Roh M. H., Liu C.-J., Laurinec S. & Margolis B. The carboxyl terminus of zona occludens-3 binds and recruits a mammalian homologue of discs lost to tight junctions. J. Biol. Chem. 277, 27501–27509 (2002). 12021270 10.1074/jbc.M201177200

[49] Sasaki T., Takagi M. & Yanagisawa T. in Ciba Foundation Symposium 205-Dental Enamel: Dental Enamel: Ciba Foundation Symposium 205. 32–53 (Wiley Online Library).

[50] Bawden J. W. Calcium transport during mineralization. The Anatomical Record 224, 226–233 (1989). 2672887 10.1002/ar.1092240212

[51] Bryant D. M. A molecular switch for the orientation of epithelial cell polarization. Dev. Cell 31, 171–187 (2014). 25307480 10.1016/j.devcel.2014.08.027 PMC4248238

[52] Wang A. Z., Ojakian G. K. & Nelson W. J. Steps in the morphogenesis of a polarized epithelium. I. Uncoupling the roles of cell-cell and cell-substratum contact in establishing plasma membrane polarity in multicellular epithelial (MDCK) cysts. J. Cell Sci. 95, 137–151 (1990). 2351699 10.1242/jcs.95.1.137

[53] Drubin D. G. & Nelson W. J. Origins of cell polarity. Cell 84, 335–344 (1996). 8608587 10.1016/s0092-8674(00)81278-7

[54] Liu F. & Millar S. Wnt/β-catenin signaling in oral tissue development and disease. J. Dent. Res. 89, 318–330 (2010). 20200414 10.1177/0022034510363373 PMC3140915

[55] Tamura M. & Nemoto E. Role of the Wnt signaling molecules in the tooth. Jpn. Dent. Sci. Rev. 52, 75–83 (2016). 28408959 10.1016/j.jdsr.2016.04.001 PMC5390339

[56] Fan L. Constitutive activation of β-catenin in ameloblasts leads to incisor enamel hypomineralization. J. Mol. Histol. 49, 499–507 (2018). 30066216 10.1007/s10735-018-9788-x

[57] Yang Y. GSK3β regulates ameloblast differentiation via Wnt and TGF-β pathways. J. Cell. Physiol. 233, 5322–5333 (2018). 29215720 10.1002/jcp.26344

[58] Liu J. Wnt/β-catenin signalling: function, biological mechanisms, and therapeutic opportunities. Signal transduction and targeted therapy 7, 3 (2022). 34980884 10.1038/s41392-021-00762-6 PMC8724284

[59] Ito Y. Overexpression of Smad2 reveals its concerted action with Smad4 in regulating TGF-β-mediated epidermal homeostasis. Dev. Biol. 236, 181–194 (2001). 11456453 10.1006/dbio.2001.0332

[60] Goodwin A. F. Abnormal Ras signaling in Costello syndrome (CS) negatively regulates enamel formation. Hum. Mol. Genet. 23, 682–692 (2014). 24057668 10.1093/hmg/ddt455 PMC3888259

[61] Otsu K. & Harada H. Rho GTPases in ameloblast differentiation. Jpn. Dent. Sci. Rev. 52, 32–40 (2016). 28408954 10.1016/j.jdsr.2015.09.001 PMC5382790

[62] Xue H. Ameloblasts require active R ho A to generate normal dental enamel. Eur. J. Oral Sci. 121, 293–302 (2013). 23841780 10.1111/eos.12059 PMC3711190

[63] Otsu K., Kishigami R., Fujiwara N., Ishizeki K. & Harada H. Functional role of rho-kinase in ameloblast differentiation. J. Cell. Physiol. 226, 2527–2534 (2011). 21792909 10.1002/jcp.22597

[64] Otsu K., Sakano M., Masuda T., Fujiwara N. & Harada H. The role of Rho-kinases in ameloblast differentiation. J. Oral Biosci. 55, 159–164 (2013).

[65] Otsu K., Ida-Yonemochi H., Fujiwara N. & Harada H. The semaphorin 4D-RhoA-Akt signal cascade regulates enamel matrix secretion in coordination with cell polarization during ameloblast differentiation. Journal of Bone and Mineral Research 31, 1943–1954 (2016). 27218883 10.1002/jbmr.2876

[66] Humphries M. J. Cell adhesion assays. Extracellular Matrix Protocols: Second Edition, 203–210 (2009). 10.1007/978-1-59745-413-1_14 19247616

[67] Lee G. Y., Kenny P. A., Lee E. H. & Bissell M. J. Three-dimensional culture models of normal and malignant breast epithelial cells. Nat. Methods 4, 359–365 (2007). 17396127 10.1038/nmeth1015 PMC2933182

